# Nationwide case–control study of risk factors and outcomes for community-acquired sepsis

**DOI:** 10.1038/s41598-021-94558-x

**Published:** 2021-07-23

**Authors:** Ann-Charlotte Lindström, Mikael Eriksson, Johan Mårtensson, Anders Oldner, Emma Larsson

**Affiliations:** 1 Perioperative Medicine and Intensive Care, Karolinska University Hospital Solna, 171 76 Stockholm, Sweden; 2 Department of Anaesthesia, Operation and Intensive Care, Uppsala University Hospital, Uppsala, Sweden; 3 Section of Anaesthesiology and Intensive Care Medicine, Department of Physiology and Pharmacology, Karolinska Institutet, Stockholm, Sweden

**Keywords:** Epidemiology, Risk factors, Comorbidities, Infectious diseases

## Abstract

Sepsis is the main cause of death in the intensive care units (ICU) and increasing incidences of ICU admissions for sepsis are reported. Identification of patients at risk for sepsis and poor outcome is therefore of outmost importance. We performed a nation-wide case–control study aiming at identifying and quantifying the association between co-morbidity and socio-economic factors with intensive care admission for community-acquired sepsis. We also explored 30-day mortality. All adult patients (n = 10,072) with sepsis admitted from an emergency department to an intensive care unit in Sweden between 2008 and 2017 and a control population (n = 50,322), matched on age, sex and county were included. In the sepsis group, 69% had a co-morbid condition at ICU admission, compared to 31% in the control group. Multivariable conditional logistic regression analysis was performed and there was a large variation in the influence of different risk factors associated with ICU-admission, renal disease, liver disease, metastatic malignancy, substance abuse, and congestive heart failure showed the strongest associations. Low income and low education level were more common in sepsis patients compared to controls. The adjusted OR for 30-day mortality for sepsis patients was 132 (95% CI 110–159) compared to controls.

## Introduction

In recent years increasing incidences of hospitalisations and intensive care admissions for sepsis have been reported^1–5^. Possible explanatory factors include an aging population, altered co-morbidity patterns, increased use of immunomodulatory drugs, invasive procedures, and emerging multi-drug resistant pathogens^6^. Sepsis is a devastating disease and the main cause of death in patients treated in intensive care units (ICU)^7^. Studies on mortality in ICU-treated sepsis patients have presented declining or unchanged trends over time^2,4,8,9^. Nevertheless, mortality rates remain high for sepsis and septic shock despite increased attention and awareness^10–12^. Identification of patients at risk for sepsis and poor outcome is of outmost importance. Demography and comorbidity in sepsis patients include a majority of males, high age, heart disease, chronic obstructive pulmonary disease, diabetes and cancer^12–14^. In accordance with other medical conditions, a linkage between socio-economic status and outcome has been presented also for sepsis patients^15,16^. Although characteristics of sepsis patients have been identified, there is a lack of evidence distinguishing factors associated with the risk of sepsis and their magnitude. We therefore performed a population-based case–control study to identify and quantify the association of co-morbidity and socio-economic factors with the risk of ICU admission for community-acquired sepsis. In addition, we wanted to analyse the influence of exposure to sepsis on short-term mortality.

## Methods

The study was approved by the regional ethical review board in Stockholm, Sweden (approval number 2018/725-31/2) and waived requirement for informed consent. The study adhered to the STROBE (Strengthening the Reporting of Observational Studies in Epidemiology) guidelines for case–control studies^17^. All research was conducted in accordance with national guidelines and regulations.

### Identification of sepsis patients and controls

Public health care, including intensive care, is tax-funded and available for all citizens in Sweden regardless of private health insurance. All residents receive a unique personal identity number at birth or upon immigration. Through this number data from nationwide registers can be linked with virtually no loss of follow-up^18^.

Community-acquired sepsis was defined as a patient with a sepsis diagnosis, admitted to the ICU directly from an emergency department. All admissions to ICUs in Sweden from January 1 2008, to December 31 2017, with either a primary diagnosis of sepsis (ICD-codes A41.9, R57.2, and R65.1), or a primary diagnosis of an infectious disease (e.g., bacterial meningitis) with sepsis as a secondary diagnosis and admitted to the ICU from the emergency department were identified through the Swedish Intensive Care Register (SIR)^19^. SIR collects individual patient data from non-neonatal Swedish intensive care units and operates within the legal framework of the Swedish National Quality Registries^20^. Written informed consent is not required, but patients may withdraw their data from the registry at any time. For patients with several admissions, only the first episode was included in the current study. Estimated Mortality Rate (EMR) was based on APACHE 2 (Acute Physiology and Chronic Health Evaluation 2) for patients included until 2012 and thereafter based on SAPS III (Simplified Acute Physiology Score III). For each patient with sepsis, the government agency Statistics Sweden selected five controls from the general population^21^. Controls were matched on age, sex, and county at the time of ICU admission for the corresponding sepsis patient.

### Exposure

Information on co-morbidities was obtained from the Swedish National Patient Register^22^. The register is managed by the National Board of Health and Welfare (NBHW) and holds information on in-patient and out-patient care episodes including ICD-10 codes. Primary care is not included in the register. Co-morbidity was assessed five years prior to ICU admission. Somatic co-morbidity was classified in accordance with Charlson’s co-morbidity Index (CCI)^23^. In addition, kidney disease was further classified as dependency on renal dialysis or not. Psychiatric illness and substance abuse were defined as the presence of a diagnosis in ICD group F20-F99 and F10-F14, F16 and F18-F19 respectively. Data on income and education for sepsis patients and controls was extracted from the Longitudinal Integration Database for Health Insurance and Labour Market Studies (LISA), managed by Statistics Sweden^24^. Level of education at the time of ICU admission was categorized as ≤ 9 years, 10–12 years, and > 12 years of schooling respectively, the last category equalling university level. Income was categorized into three groups: low, moderate and high. Low income was defined as an income < 50%, and high income as an income ≥ 200% of the median income the calendar year before ICU admission. Information on mortality were obtained from the Cause of Death Register^25^.

### Statistical analysis

Characteristics of the study cohort are presented as proportions and percentages for categorical data. Continuous data are presented as median with interquartile ranges (IQR) or mean with standard deviations (SD) where applicable. Associations between potential risk factors and sepsis were estimated by conditional logistic regression and expressed as odds ratios (OR) with corresponding 95% confidence intervals (CI). Covariates with univariate significance were carried forward to the multivariable models. Odds ratios from the univariate and multivariable regression models are presented to express the likelihood of being admitted to ICU with sepsis. All analyses were carried out for the entire study cohort as well as separately for patients with no registered somatic co-morbidity at the time of ICU admission. Finally, the association between sepsis and 30-day mortality were explored using logistic regression. Data were analysed as complete cases. A two-sided *p* value < 0.05 was considered statistically significant. Stata/MP 14.2 (StataCorp, College Station, TX) was used for all analyses.

## Results

### Demography and 30-day mortality

The study population comprised 10 072 sepsis patients and 50 322 controls. Characteristics of the study cohort are presented in Table 1. There was male dominance (58%) and the mean age was 67 years. More than half of the sepsis patients had a CCI of ≥ 2 compared to 18% in the control group. The most common comorbidities among the sepsis patients were congestive heart failure, diabetes, and chronic obstructive pulmonary disease (COPD), whereas 31% of the sepsis patients had no reported somatic co-morbidity on admission. Immunosuppression therapy, psychiatric illness and substance abuse were more common among sepsis patients. High levels of income and education were more common in control individuals compared to sepsis patients. The 30-day mortality of 27% was in agreement with the median EMR of 0.28, but a 100-fold higher for septic patients as compared with controls. Missing values were noted for education (1.8%) and income (0.4%).

**Table 1:** Baseline characteristics.

Characteristics	Sepsis patients (n = 10,072)	Controls (n = 50,322)
Females, n (%)	4209 (41.8)	21,045 (41.8)
**Age, y**		
Mean (SD)	67.1 (15.1)	67.1 (15.1)
Median (IQR)	70 (60–78)	70 (60–78)
Range	18–98	18–98
**Level of education, n (%)**		
≤ 9 years	4107 (40.8)	16,731 (33.2)
10–12 years	4105 (40.8)	20,473 (40.7)
> 12 years	1637 (16.3)	12,273 (24.3)
**Income, n (%)**		
Low	1412 (14.0)	5720 (11.4)
Moderate	8250 (81.9)	41,063 (81.6)
High	382 (3.8)	3330 (6.6)
Co-morbidity, n (%)	6961 (69.1)	15,445 (30.7)
**CCI categories, n (%)**		
0	3111 (30.9)	34,877 (69.3)
1	1646 (16.3)	6257 (12.4)
≥ 2	5315 (52.8)	9188 (18.3)
Myocardial infarction	1214 (12.1)	2775 (5.5)
Congestive heart failure	1898 (18.8)	2413 (4.8)
COPD	1622 (16.1)	2430 (4.8)
Peripheral vascular disease	896 (8.9)	1210 (2.4)
Cerebrovascular disease	1314 (13.0)	3037 (6.0)
**Liver disease**		
Mild	443 (4.4)	302 (0.6)
Moderate	220 (2.2)	67 (0.1)
**Diabetes**		
Without complications	1351 (13.4)	2795 (5.6)
With complications	921 (9.1)	1289 (2.6)
**Kidney disease**		
Moderate	823 (8.2)	823 (1.6)
ESRD	85 (0.8)	23 (0.0)
**Malignancy**		
Non-metastatic	1503 (14.9)	3805 (7.6)
Metastatic	529 (5.3)	536 (1.1)
Immunosuppressive therapy	1838 (18.2)	1496 (3.0)
Psychiatric illness	1534 (15.2)	2635 (5.2)
Substance abuse	939 (9.3)	812 (1.6)
**Mortality, n (%)**		
30-day	2675 (26.6)	128 (0.3)
EMR, median (IQR)	0.28 (0.14–0.47)	

*SD* Standard deviation, *IQR* Interquartile range, *CCI* Charlson’s comorbidity index, *COPD* Chronic obstructive pulmonary disease, *ESRD* End-stage renal disease, *EMR* Estimated mortality rate.

For the subset of sepsis patients without somatic co-morbidity on admission psychiatric illness, substance abuse, as well as low income and education were more common as compared with controls. Also, in this subset of sepsis patients the 30-day mortality was notably increased compared to controls, 20% versus 0.2%, Table 2.

**Table 2:** Baseline characteristics of sepsis patients without somatic comorbidity at ICU admission and controls.

Characteristics	Sepsis patients (n = 3111)	Controls (n = 15,544)
Females, n (%)	1390 (44.7)	6950 (44.7)
**Age, y**		
Mean (SD)	61.5 (17.6)	61.4 (17.6)
Median (IQR)	64 (50–75)	64 (50–75)
Range, min–max	18–98	18–98
**Level of education, n (%)**		
≥ 9 years	1150 (37.0)	4459 (28.7)
10–12 years	1285 (41.3)	6657 (42.8)
> 12 years	570 (18.3)	4161 (26.8)
**Income, n (%)**		
Low	492 (15.8)	1913 (12.3)
Moderate	2462 (79.1)	12,484 (80.3)
High	132 (4.2)	1081 (7.0)
Psychiatric illness	420 (13.5)	896 (5.8)
Substance abuse	242 (7.8)	270 (1.7)
**Mortality, n (%)**		
30-day	625 (20.3)	28 (0.2)
90-day	728 (23.6)	86 (0.6)
1-year	841 (27.3)	362 (2.3)
EMR, median (IQR)	0.21 (0.09–0.4)	

*ICU* Intensive care unit, *SD* Standard deviation, *EMR* Estimated mortality rate.

### Risk of sepsis

**Figure 1:**
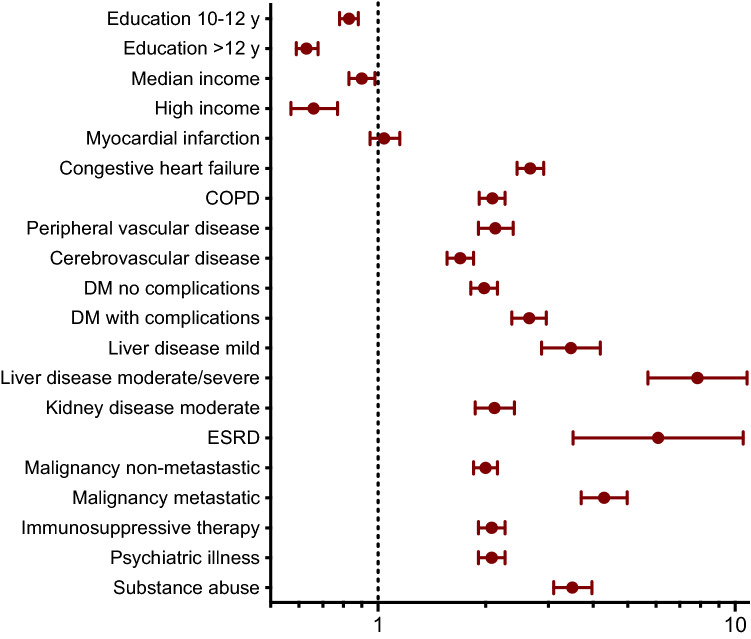
Multivariable logistic regression analysis of risk factors for community-acquired sepsis. Logarithmic scale for odds ratios with 95% confidence intervals on the x-axis. *COPD* Chronic obstructive pulmonary disease, *DM* Diabetes mellitus, *ESRD* End-stage renal disease.

In the univariate conditional logistic regression of the total cohort, all co-morbid conditions as well as low income and low education were significantly associated with the risk of ICU admission for sepsis Table 3. In the multivariable analysis, all covariates apart from myocardial infarction were still associated with sepsis. There were notable differences in the contribution of individual risk factors, the strongest association was observed for end-stage renal disease (ESRD), liver disease, metastatic malignancy, substance abuse and congestive heart failure, Fig. 1.

**Table 3:** Logistic regression analysis for the risk of community-acquired sepsis, all patients.

	Univariate		Multivariable	
	OR (95% CI)	*p* value	OR (95% CI)	*p* value
**Level of education**				
≤ 9 years	Ref		Ref	
10–12 years	0.78 (0.74–0.82)	< 0.001	0.83 (0.78–0.88)	< 0.001
> 12 years	0.51 (0.47–0.54)	< 0.001	0.63 (0.59–0.68)	< 0.001
**Income**				
Low	Ref		Ref	
Moderate	0.79 (0.74–0.84)	< 0.001	0.90 (0.83–0.98)	0.014
High	0.43 (0.39–0.50)	< 0.001	0.66 (0.57–0.77)	< 0.001
**Comorbidity**				
Myocardial infarction	2.45 (2.28–2.64)	< 0.001	1.04 (0.95–1.15)	0.382
Congestive heart failure	5.23 (4.87–5.60)	< 0.001	2.67 (2.45–2.91)	< 0.001
COPD	3.88 (3.62–4.16)	< 0.001	2.09 (1.92–2.27)	< 0.001
Peripheral vascular disease	4.09 (3.73–4.48)	< 0.001	2.13 (1.91–2.39)	< 0.001
Cerebrovascular disease	2.45 (2.28–2.63)	< 0.001	1.70 (1.56–1.85)	< 0.001
**Liver disease**				
No	Ref		Ref	
Mild	7.91 (6.80–9.20)	< 0.001	3.47 (2.87–4.19)	< 0.001
Moderate/severe	17.38 (13.17–22.95)	< 0.001	7.84 (5.70–10.79)	< 0.001
**Diabetes**				
No	Ref		Ref	
Without complications	2.98 (2.78–3.20)	< 0.001	1.98 (1.82–2.16)	< 0.001
With complications	4.37 (3.99–4.77)	< 0.001	2.65 (2.37–2.96)	< 0.001
**Kidney disease**				
No	Ref		Ref	
Moderate	5.63 (5.08–6.23)	< 0.001	2.12 (1.87–2.41)	< 0.001
ESRD	19.28 (12.15–30.60)	< 0.001	6.09 (3.52–10.54)	< 0.001
**Malignancy**				
No	Ref		Ref	
Non-metastatic	2.35 (2.20–2.51)	< 0.001	2.00 (1.85–2.16)	< 0.001
Metastatic	5.74 (5.07–6.49)	< 0.001	4.30 (3.71–4.99)	< 0.001
Immunosuppressive therapy	7.40 (6.87–7.97)	< 0.001	2.08 (1.91–2.27)	< 0.001
Psychiatric illness	3.33 (3.11–3.57)	< 0.001	2.08 (1.91–2.27)	< 0.001
Substance abuse	6.55 (5.93–7.24)	< 0.001	3.50 (3.10–3.97)	< 0.001

*OR* Odds ratio, *CI* Confidence interval, *COPD* Chronic obstructive pulmonary disease, *ESRD* End-stage renal disease.

For patients with no registered somatic co-morbidity on admission, psychiatric illness and substance abuse were significantly associated with the risk of sepsis. Individuals with high education had a lower risk (OR 0.55, 95% CI 0.49–0.62) of sepsis compared to those with low education. High level of income was also significantly associated with a lower risk of sepsis compared to low income (OR 0.62, 95% CI 0.50–0.78) Table 4.

**Table 4:** Logistic regression analysis for the risk of community-acquired sepsis, patients with no somatic comorbidity.

	Univariate		Multivariable	
	OR (95% CI)	*p* value	OR (95% CI)	*p* value
**Level of education**				
≥ 9 years	Ref		Ref	
10–12 years	0.70 (0.64–0.77)	< 0.001	0.75 (0.68–0.82)	< 0.001
> 12 years	0.48 (0.43–0.54)	< 0.001	0.55 (0.49–0.62)	< 0.001
**Income**				
Low	Ref		Ref	
Moderate	0.73 (0.65–0.82)	< 0.001	0.83 (0.73–0.94)	0.003
High	0.44 (0.35–0.54)	< 0.001	0.62 (0.50–0.78)	< 0.001
Psychiatric illness	2.61 (2.30–2.96)	< 0.001	1.89 (1.65–2.17)	< 0.001
Substance abuse	4.87 (4.06–5.84)	< 0.001	3.50 (2.88–4.26)	< 0.001

*OR* Odds ratio, *CI* Confidence interval.

### Risk of 30-day mortality

After adjustment for somatic co-morbidity, socio-economy, psychiatric co-morbidity and substance abuse, exposure to ICU admission for sepsis was strongly associated with 30-day mortality with an OR of 132 (95% CI 110–159) compared with controls. For the subset of patients without prior somatic co-morbidity the risk of 30-day mortality was even higher with an OR of 151 (95% CI 103–221), even after adjustment for substance abuse, socio-economy and psychiatric illness.

## Discussion

This is to the best of our knowledge one of the largest nationwide studies aiming at studying ICU-treated patients with community-acquired sepsis. In this large case–control study we found several factors associated with ICU-admission for community-acquired sepsis. There was a large variation in the contribution of individual risk factors, where ESRD, liver disease, metastatic malignancy, substance abuse and congestive heart failure were the strongest. For patients without any somatic co-morbidity prior to ICU admission, a history of substance abuse, psychiatric illness and low socio-economy were associated with ICU admission for sepsis. The adjusted risk of 30-day mortality was increased by more than 100-fold for sepsis patients compared to controls. This was also seen in patients without prior somatic co-morbidity.

Several risk-factors for sepsis identified in our study are well-recognized in the literature. The magnitude of their respective influence on the risk of sepsis are, however, not fully elucidated. Malignancy and immunosuppression are closely linked entities that significantly increase the risk of severe infections. In a large Danish study, the odds ratio of hospital admission for sepsis was 1.4 and 4.4 for cancer and immunosuppression respectively^26^. Diabetes is commonly advocated as a risk factor for sepsis^26,27^. It is proposed that diabetes causes a functional immune deficiency reducing immune cell function^28^. Moreover, diabetic patients commonly develop complications like chronic ulcers, renal disease and angiopathy that may further increase the risk of infections. This was seen in the current study where the risk of sepsis was higher in diabetics patients with complications. Cardiovascular disease as an entity is reported to increase the risk of sepsis^26^. In our study, congestive heart failure increased the risk of sepsis significantly whereas myocardial infarction did not. The last finding contrasts to a previous report where a history of myocardial infarction almost doubled the risk of hospitalisation for sepsis. This American study was based on data between 2003 and 2011 and the difference noted may reflect more elaborated coronary care systems during the last decade^27^. A recent UK study showed that sepsis was the cause of death in almost a quarter of congestive heart failure patients dying within a mean follow-up time of 4 years^29^. As such it was the second most common cause of death after progressive heart failure. This unexpectedly high figure may be due to a limited cardiovascular reserve and the fact that heart failure may affect the immune system per se which increases the risk of an infection progressing to a septic state. These findings are well in line with the current study where congestive heart failure was seen in almost a fifth of the cohort and one of the strongest risk factors.

The liver plays a central role in immunological homeostasis acting as a lymphoid organ in response to sepsis^30^. In this sense, a patient with liver disease represents an immunocompromised host with an increased risk of sepsis. In addition to a general susceptibility to infections, the specific problem of spontaneous bacterial peritonitis in severe liver disease is a recognised etiology of sepsis^31^. Not unexpectedly, moderate to severe liver injury was the strongest risk factor for sepsis in our study. End-stage renal disease with dialysis treatment comprised a small group of patients with a notably high risk of ICU admission for sepsis in our study cohort. Infections are reported to be the second leading cause of death after cardiovascular disease among ESRD patients^32^. In a US study, the risk of dying from sepsis was increased several 100-fold in ESRD patients as compared with the general population^32^. These findings could in part be explained by acquired immune deficiency due to uraemia as well as repetitive exposure of patients to potential infectious risk factors during the course of dialysis therapy including repeated disruption of the skin barrier. Metastatic malignancy was an expected strong risk factor for sepsis with a number of plausible explanations including immune deficiency due to chemotherapy, radiotherapy, cancer per se and catabolism as well as exposure to invasive procedures such as surgery and diagnostic procedures. Endothelial dysfunction is an important part of septic pathophysiology and also a component of several chronic diseases^33^. This endothelial fragility may constitute a mechanism by which these co-morbidities increase the risk of sepsis as seen in the present study.

A history of substance abuse proved to be a strong risk factor for sepsis in the current study. This entity comprises several subgroups including alcohol abuse, intravenous and non-intravenous drug use. Already in the late 1700s, it was suggested that excessive use of alcohol is associated with an increased risk of infection^34^. In the early twentieth century, Sir William Osler postulated that alcohol abuse was the most potent predisposing condition for the development of bacterial pneumonia^34^. Chronic alcohol users are exposed to a number of potential risk factors such as aspiration, poor dental hygiene, reluctance to seek health care as well as immunological impairments such as reduced pulmonary phagocytic activity and neutrophil recruitment^35^. Moreover, alcohol has been reported to increase gut permeability potentially leading to translocation of bacteria and endotoxin^35^. Intravenous drug abuse is closely associated with an increased risk of infections such as endocarditis and sepsis due to repeated skin lacerations and the use of non-sterile syringes. In addition, many of the drugs used both intravenously and by oral intake have immunological effects. Opioids have been shown to be immunosuppressive by several mechanisms including down-regulation of natural killer cells^36^. Recently opioid use disorder was reported to contribute disproportionately to sepsis deaths among younger and healthier patients in the US^37^.

Relationships between socio-economic factors and outcomes have been demonstrated in several medical conditions including cancer, heart disease and stroke^16^. There are previous reports from various settings studying septic patients, but the relationship between socio-economic status and the risk of severe sepsis is not fully understood. In a report from the U.S. including more than 600 000 patients with sepsis, low household income level was associated with in-hospital mortality^16^. The authors’ estimation of income was based on the median household income level for the zip code of the patient’s residence. A Danish population-based case–control study, including more than 4000 patients demonstrated that a low socio-economic position was associated with an increased risk hospitalisation for community-acquired bacteraemia^38^. In another Danish study of 387 septic ICU patients, low income was significantly associated with increased 30-day mortality^39^. In the current study, we investigated the association between income and education and the risk of severe community-acquired sepsis. We found a markedly increased risk for patients with low income and low education, even after adjustment for somatic co-morbidities, psychiatric illness and substance abuse. While a number of factors have been suggested to increase the risk of infections in individuals with a low socio-economic position, there is a lack of evidence when it comes to causative associations. Suggested factors include lifestyle aspects such as less engagement in preventive behaviours including exercising, compliance with vaccination, nutritional status and smoking habits. Other factors may include household crowding, reluctance to seek health care and exposure to chronic stress. In order to test the robustness of the association between socio-economy and the risk for sepsis, we also performed a stratified analysis including only patients with no reported somatic co-morbidity at ICU admission. The results remained largely unchanged reflecting the importance of these factors for the risk of severe infections. For this subset of patient’s psychiatric illness and in particular, substance abuse proved to be risk factors for sepsis.

The 30-day mortality of 26.7% was on par with the estimated mortality ratio of 0.28 for septic patients. After adjustment for all included somatic co-morbid factors, substance abuse, psychiatric illness, and socio-economy, the risk of 30-day mortality was markedly higher for sepsis patients with an OR of 130. Somewhat surprisingly this figure was even higher for patients without reported somatic co-morbidity on admission. These figures undoubtedly underline the severity that comes with community-acquired sepsis deeming ICU care and the need for further knowledge of risk factors.

### Strengths/limitation

Our study has several strengths. We analysed a large, nationwide multicentre cohort of ICU-admitted sepsis patients strengthening external validity and generalisability. Data underwent a two-step validation process (at each participating ICU before submission to SIR and centrally before data extraction), assuring a high level of internal validity. The study is further strengthened by the linkages with well-validated national health registries. Demographic characteristics are much in line with previous studies of ICU sepsis patients. Limitations include the register-based design. Only patients admitted to ICU were included in the study population and no comparisons were made with hospitalised patients not admitted to ICU. We chose to include only patients admitted from the emergency department in order to minimize the heterogeneity of the sepsis population and facilitate comparisons with other populations, this inclusion criterion could obviously also limit generalisability. Misclassification of sepsis is possible in this registry-based study. However, it is unlikely that such misclassification affects the associations between comorbidities and socio-economy and the risk of sepsis.

## Conclusions

In this nationwide case–control study we identified a number of risk factors for ICU-admission for community-acquired sepsis. These factors included low socio-economy, psychiatric illness, substance abuse and all somatic co-morbid conditions included in the Charlson’s co-morbidity index apart from myocardial infarction. The influence of these risk factors was highly differentiated where ESRD, liver disease, metastatic malignancy, substance abuse and congestive heart failure were the strongest. For patients without somatic co-morbidity, low socio-economy, psychiatric illness and substance abuse were associated with sepsis. After adjustment for baseline factors, the risk of death was still markedly increased in sepsis patients. Early awareness of septic manifestations in patients with a high comorbidity burden and low socioeconomy is of outmost importance.

## Data Availability

The datasets generated and analysed during the current study are available from the corresponding author on reasonable request.

## References

[1] Kumar G (2011). Nationwide trends of severe sepsis in the 21st century (2000–2007). Chest.

[2] Shankar-Hari M, Harrison DA, Rubenfeld GD, Rowan K (2017). Epidemiology of sepsis and septic shock in critical care units: Comparison between sepsis-2 and sepsis-3 populations using a national critical care database. Br. J. Anaesth..

[3] Martin GS, Mannino DM, Eaton S, Moss M (2003). The epidemiology of sepsis in the United States from 1979 through 2000. N. Engl. J. Med..

[4] Valles J (2019). Trends in the incidence and mortality of patients with community-acquired septic shock 2003–2016. J. Crit. Care.

[5] Rubens M (2020). Increasing sepsis rates in the United States: Results from national inpatient sample, 2005 to 2014. J. Intensive Care Med.

[6] Rhee C, Klompas M (2020). Sepsis trends: Increasing incidence and decreasing mortality, or changing denominator?. J. Thorac. Dis..

[7] Fleischmann C (2016). Assessment of global incidence and mortality of hospital-treated sepsis. Current estimates and limitations. Am. J. Respir. Crit. Care Med..

[8] Kaukonen KM, Bailey M, Suzuki S, Pilcher D, Bellomo R (2014). Mortality related to severe sepsis and septic shock among critically ill patients in Australia and New Zealand, 2000–2012. JAMA.

[9] Strandberg G, Walther S, Agvald Öhman C, Lipcsey M (2020). Mortality after severe sepsis and septic shock in Swedish intensive care units 2008–2016-A nationwide observational study. Acta Anaesthesiol. Scand..

[10] Singer M (2016). The third international consensus definitions for sepsis and septic shock (Sepsis-3). JAMA.

[11] Kadri SS (2017). Estimating ten-year trends in septic shock incidence and mortality in United States academic medical centers using clinical data. Chest.

[12] Rhee C (2017). Incidence and trends of sepsis in US hospitals using clinical vs claims data, 2009–2014. JAMA.

[13] Angus DC (2001). Epidemiology of severe sepsis in the United States: Analysis of incidence, outcome, and associated costs of care. Crit. Care Med..

[14] Wilhelms SB, Walther SM, Huss F, Sjöberg F (2017). Severe sepsis in the ICU is often missing in hospital discharge codes. Acta Anaesthesiol. Scand..

[15] Welch CA, Harrison DA, Hutchings A, Rowan K (2010). The association between deprivation and hospital mortality for admissions to critical care units in England. J. Crit. Care.

[16] Rush B (2018). Association of household income level and in-hospital mortality in patients with sepsis: A nationwide retrospective cohort analysis. J. Intensive Care Med..

[17] von Elm E (2014). The strengthening the reporting of observational studies in epidemiology (STROBE) statement: Guidelines for reporting observational studies. Int. J. Surg..

[18] Ludvigsson JF, Otterblad-Olausson P, Pettersson BU, Ekbom A (2009). The Swedish personal identity number: Possibilities and pitfalls in healthcare and medical research. Eur. J. Epidemiol..

[19] *Svenska Intensivvårdsregistret (SIR)*, https://www.icuregswe.org/.

[20] *Nationella kvalitetsregister, Swedish National Quality Registries.*, https://kvalitetsregister.se/index.html.

[21] SCB. *Statistics Sweden*, https://www.scb.se/en/.

[22] Socialstyrelsen. *Patientregistret*, https://www.socialstyrelsen.se/statistik-och-data/register/alla-register/patientregistret/ (2018).

[23] Quan H (2005). Coding algorithms for defining comorbidities in ICD-9-CM and ICD-10 administrative data. Med. Care.

[24] Sweden, S. *Longitudinal integrated database for health insurance and labour market studies (LISA)*, https://www.scb.se/en/services/guidance-for-researchers-and-universities/vilka-mikrodata-finns/longitudinella-register/longitudinal-integrated-database-for-health-insurance-and-labour-market-studies-lisa/. 10.1007/s10654-019-00511-8 PMC6451717 30929112

[25] Socialstyrelsen, T. S. b. o. h. a. w. *Dödsorsaksregistret, Cause of death register*, https://www.socialstyrelsen.se/statistik-och-data/register/alla-register/dodsorsaksregistret/ (2018).

[26] Henriksen DP (2015). Risk factors for hospitalization due to community-acquired sepsis—A population-based case-control study. PLoS ONE.

[27] Wang HE (2012). Chronic medical conditions and risk of sepsis. PLoS ONE.

[28] Frydrych LM, Fattahi F, He K, Ward PA, Delano MJ (2017). Diabetes and sepsis: Risk, recurrence, and ruination. Front. Endocrinol. (Lausanne).

[29] Walker AMN (2018). Prevalence and predictors of sepsis death in patients with chronic heart failure and reduced left ventricular ejection fraction. J. Am. Heart Assoc..

[30] Yan J, Li S, Li S (2014). The role of the liver in sepsis. Int. Rev. Immunol..

[31] Bunchorntavakul C, Chamroonkul N, Chavalitdhamrong D (2016). Bacterial infections in cirrhosis: A critical review and practical guidance. World J. Hepatol..

[32] Sarnak MJ, Jaber BL (2000). Mortality caused by sepsis in patients with end-stage renal disease compared with the general population. Kidney Int..

[33] Bermejo-Martin JF, Martín-Fernandez M, López-Mestanza C, Duque P, Almansa R (2018). Shared features of endothelial dysfunction between sepsis and its preceding risk factors (aging and chronic disease). J. Clin. Med..

[34] Moss M (2005). Epidemiology of sepsis: race, sex, and chronic alcohol abuse. Clin. Infect. Dis..

[35] Trevejo-Nunez G, Kolls JK, de Wit M (2015). Alcohol use as a risk factor in infections and healing: A clinician’s perspective. Alcohol Res..

[36] Eisenstein TK (2019). The role of opioid receptors in immune system function. Front. Immunol..

[37] Alrawashdeh M, Klompas M, Kimmel S (2020). 56: Epidemiology, outcomes, and trends of sepsis in patients with opioid use disorders in U.S. hospitals. Crit. Care Med..

[38] Koch K, Søgaard M, Nørgaard M, Thomsen RW, Schønheyder HC (2014). Socioeconomic inequalities in risk of hospitalization for community-acquired bacteremia: A Danish population-based case-control study. Am. J. Epidemiol..

[39] Schnegelsberg A (2016). Impact of socioeconomic status on mortality and unplanned readmission in septic intensive care unit patients. Acta Anaesthesiol. Scand..

